# Four-electron conversion vanadium-based battery

**DOI:** 10.1093/nsr/nwag437

**Published:** 2026-07-20

**Authors:** Jie Chen, Fabrizia Foglia, Ivan P Parkin, Guanjie He

**Affiliations:** Department of Chemistry, University College London, UK; Department of Chemistry, University College London, UK; Department of Chemistry, University College London, UK; Department of Chemistry, University College London, UK

Vanadium-based materials have attracted broad attention in battery research because of their low cost and high specific capacity. In aqueous batteries, they typically exhibit well-defined redox pathways together with favourable rate performance and stability at high current densities [1–3]. Consequently, vanadium-based materials have become widely adopted electrode materials in recent reports of Ah-scale aqueous zinc batteries [4–6].

Vanadium-based materials commonly feature relatively open crystal frameworks, with layered or tunnel-type structures, and store energy predominantly through ion insertion and extraction. However, the finite availability of crystallographic insertion sites limits their charge-storage capacity. In addition, in near-neutral electrolytes, the redox potentials of many vanadium-based insertion materials are concentrated around 0.1 V versus the standard hydrogen electrode (SHE), limiting the attainable cell voltage whether they are used as cathodes or anodes [7].

**Figure 1.**
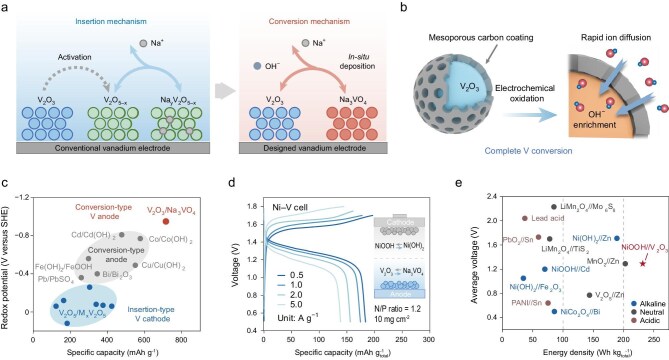
(a) Schematic illustration of the insertion mechanism of V_2_O_3_ electrodes in near-neutral media and the conversion mechanism in alkaline media. (b) Schematic illustration of the electrochemical oxidation process in mesoC@V_2_O_3_. (c) Comparison of the V_2_O_3_/Na_3_VO_4_ anode with representative vanadium-based cathodes and conversion-type anodes. Blue and grey shading denote the approximate electrochemical performance windows of insertion-type vanadium-based cathodes and reported conversion-type anodes, respectively. (d) Galvanostatic voltage profiles of the Ni-V cell with an N/P ratio of 1.2 under different current densities. Inset, schematic illustration of the alkaline Ni-V cell configuration, comprising a mesoC@V_2_O_3_ anode and an NiOOH cathode. The specific capacity is calculated based on the total electrode mass. (e) Comparison of average discharge voltage and energy density between the alkaline Ni-V cell and conventional aqueous battery systems. Reproduced from ref. [8] with permission.

A recent study by Jin *et al*. reported, for the first time, a conversion-type vanadium redox chemistry and introduced a V_2_O_3_ anode capable of four-electron transfer. Specifically, Na^+^-induced *in situ* precipitation enabled the reversible transformation between V_2_O_3_ and Na_3_VO_4_, and hydroxide activation converted the charge-storage pathway of V_2_O_3_ from conventional ion insertion to a reversible conversion reaction (Fig. 1a) [8].

The extensive consumption of OH^−^ during V_2_O_3_ oxidation induces local pH fluctuations, which can limit the extent of vanadium conversion and overall material utilization. To address this issue, the authors exploited the ion-transport advantages of mesoporous structures and constructed a mesoporous microreactor to stabilize the local pH during the redox process. Multiple operando synchrotron-based characterizations showed that the tailored mesoporous carbon-coated V_2_O_3_ architecture, denoted as mesoC@V_2_O_3_, enriches local OH^−^ and facilitates the reversible conversion between V_2_O_3_ and Na_3_VO_4_ (Fig. 1b).

The V_2_O_3_/Na_3_VO_4_ redox couple offers a high theoretical capacity of 715 mAh g^−^^1^ and a low redox potential of −0.95 V versus SHE, distinguishing it from conventional vanadium-based cathode materials commonly used in metal-ion batteries. Compared with advanced anode materials based on bismuth, copper and iron, the conversion-type V_2_O_3_ anode also exhibits advantages in both redox potential and specific capacity (Fig. 1c).

At the device level, the authors paired the mesoC@V_2_O_3_ anode with a commercial NiOOH cathode to construct an alkaline nickel-vanadium battery. At an N/P ratio of 1.2 and an anode loading of 10 mg cm^−^^2^, the nickel-vanadium battery delivered 183 mAh g^−^^1^ based on the combined mass of both electrodes, with an average discharge voltage of 1.29 V (Fig. 1d). The full cell achieved an energy density of 232 Wh kg^−^^1^ and showed competitive performance compared with aqueous storage systems such as lead-acid batteries, neutral manganese-zinc batteries and alkaline nickel-zinc batteries (Fig. 1e).

Overall, Dr. Chao as a pioneer in exploring new redox reaction for aqueous batteries, has helped advance the field beyond conventional ion-insertion chemistry towards the rational design of high-energy redox pathways. This work embodies this concept by revealing OH^−^ as an active chemical regulator that steers vanadium redox chemistry from ion insertion to a multielectron conversion reaction [9]. More broadly, it highlights the value of understanding and optimizing energy-storage behaviour from a chemical perspective [10], offering a general strategy for using electrolyte species to reshape reaction pathways and unlock high-energy redox chemistry in aqueous batteries and related storage systems.
